# Biomechanical effects of transverse connectors on total en bloc spondylectomy of the lumbar spine: a finite element analysis

**DOI:** 10.1186/s13018-023-03977-1

**Published:** 2023-07-05

**Authors:** Ye Han, Xuehong Ren, Yijie Liang, Xiaoyong Ma, Xiaodong Wang

**Affiliations:** 1 Department of Orthopaedics, Affiliated Hospital of Hebei University, No. 212, Yuhua Road, Hebei Baoding City, 071000 China; 2 Hebei University, Hebei Baoding City, China

**Keywords:** Biomechanics, Transverse connector, Finite element analysis, Spinal surgery, Total en bloc spondylectomy

## Abstract

**Background:**

The influence of total en bloc spondylectomy (TES) on spinal stability is substantial, necessitating strong fixation to restore spinal stability. The transverse connector (TC) serves as a posterior spinal instrumentation that connects the left and right sides of the pedicle screw-rod system. Several studies have highlighted the potential of a TC in enhancing the stability of the fixed segments. However, contradictory results have suggested that a TC not only fails to improve the stability of the fixed segments but also might promote stress associated with internal fixation. To date, there is a lack of previous research investigating the biomechanical effects of a TC on TES. This study aimed to investigate the biomechanical effects of a TC on internal fixation during TES of the lumbar (L) spine.

**Methods:**

A single-segment (L3 segment) TES was simulated using a comprehensive L spine finite element model. Five models were constructed based on the various positions of the TC, namely the intact model (L1-sacrum), the TES model without a TC, the TES model with a TC at L1–2, the TES model with a TC at L2–4, and the TES model with a TC at L4–5. Mechanical analysis of these distinct models was conducted using the Abaqus software to assess the variations in the biomechanics of the pedicle screw-rod system, titanium cage, and adjacent endplates.

**Results:**

The stability of the surgical segments was found to be satisfactory across all models. Compared with the complete model, the internal fixation device exhibited the greatest constraint on overextension (95.2–95.6%), while showing the least limitation on left/right rotation (53.62–55.64%). The application of the TC had minimal effect on the stability of the fixed segments, resulting in a maximum reduction in segment mobility of 0.11° and a variation range of 3.29%. Regardless of the use of a TC, no significant changes in stress were observed for the titanium cage. In the model without the TC, the maximum von Mises stress (VMS) for the pedicle screw-rod system reached 136.9 MPa during anterior flexion. Upon the addition of a TC, the maximum VMS of the pedicle screw-rod system increased to varying degrees. The highest recorded VMS was 459.3 MPa, indicating a stress increase of 335.5%. Following the TC implantation, the stress on the adjacent endplate exhibited a partial reduction, with the maximum stress reduced by 27.6%.

**Conclusion:**

The use of a TC in TES does not improve the stability of the fixed segments and instead might result in increased stress concentration within the internal fixation devices. Based on these findings, the routine utilisation of TC in TES is deemed unnecessary.

## Introduction

Total en bloc spondylectomy (TES) has demonstrated efficacy in treating primary spinal tumours and metastases [1, 2]. However, TES involves the complete removal of the anterior and posterior spinal structures, resulting in severe spinal instability. Therefore, a 360° reconstruction is required in TES to restore spinal stability. Complications following TES have been reported at a relatively high rate, ranging from 12 to 40% [3–6]. Failure of internal fixation is one of the most common complications after TES, with increased risk due to spinal instability. Internal fixation failure often manifests as pain, deformity, and worsening of neurological symptoms, significantly affecting the patient's quality of life. Enhancing spinal stability following TES can effectively reduce the incidence of internal fixation failure, facilitating successful bony fusion.

Several studies have indicated that the use of transverse connectors (TCs), which are posterior fixation devices connecting pedicle screw-rod systems, might enhance the stability of internal fixation [7–10]. However, it has also been observed that TCs could lead to stress concentration in the internal fixation, thereby increasing the incidence of internal fixation failure [11]. Moreover, the application of TCs could contribute to higher medical expenses. Consequently, there is a need to investigate the biomechanical effects of TCs on internal fixation in TES. Such research has the potential to enhance surgical protocols, reduce surgery-related complications, and minimise medical costs. Currently, no studies have examined whether TCs should be used in TES. This study aims to compare the range of motion (ROM), the pedicle screw-rod system, and the stress on the titanium cage between different finite element models using finite element analysis (FEA) techniques. The objective is to evaluate the superiority of various surgical methods and provide clinicians with a theoretical basis for developing surgical plans.

Based on our rationale, it was postulated that placement of a TC during TES of the lumbar (L) spine might not improve the overall stability of the fixation. Instead, it could potentially lead to increased stress concentration within the internal fixation devices.

## Materials and methods

### Study participants

The trial was performed at the Affiliated Hospital of Hebei University. A single male volunteer, aged 21 years, with a height of 175 cm and weight of 64 kg, and no history of spinal diseases, underwent computed tomography (CT) scanning of the spinal segments L1-sacrum. The CT scan produced 343 images with a 1-mm layer thickness, which were then imported in the Digital Imaging and Communications in Medicine (DICOM) format into the medical image processing software, Mimics 21.0 (MATERIALISE Inc. Leuven, Belgium). The images were processed using Mimics 21.0, 3-matic Research 13.0 (MATERIALISE Inc.), Geomagic Studio 2017 (3D Systems, Inc., Rock Hill, SC, USA), and HyperMesh2017 (Altair Engineering, Troy, MI, USA) for three-dimensional reconstruction, structural partitioning, and finite element pre-processing of the spinal segments L1-sacrum model. Subsequently, finite element processing was conducted in Abaqus 2019 (Abaqus Inc., USA).

### Construction of a complete FEA model (L1-sacrum)

First, the medical images were imported into Mimics 21.0 software in DICOM format, enabling the construction of the preliminary model representing the spinal segments L1-S. Following this, a simple smoothing process was applied to refine the model. Subsequently, the model was transferred to 3-matic Research 13.0, where the bone structure of the spine was classified into cortical bone, cancellous bone, and posterior structures, based on the physiological structure of the human body. Furthermore, the intervertebral disc was established, encompassing superior and inferior endplates, annulus fibrosus, nucleus pulposus, and facet joints. These corresponding structures underwent refinement and smoothing. Finally, the model was exported in the.stl format and subsequently imported into Geomagic. The model underwent precise surfacing, involving the detection of contour lines, construction of surface patches, and lattice construction. Subsequently, the surface was fitted to obtain a preliminary solid model. The model was then exported in.igs format and imported into HyperMesh 2017. The intervertebral disc, vertebral body, and facet joint were meshed. Furthermore, the nucleus pulposus and annulus fibrosus were intersected to simulate the annulus fibrosus fibres. Additionally, the simulation included seven ligaments, namely the anterior longitudinal ligament (ALL), posterior longitudinal ligament (PLL), ligamentum flavum (LF), interspinous ligament (ISL), supraspinous ligament (SSL), transverse ligament (TL), and capsule ligament (CL). Distinct material properties were assigned to different components, including the vertebral body, intervertebral disc, and facet joint. Finally, the model was exported in the.inp format.

The Abaqus 2019 software was used to import and assemble the.inp file. The model included various components, such as the intervertebral disc, vertebral body, and facet joints, and their interactions were accurately modelled. The corresponding mechanical properties were assigned for FEA. Tetrahedral elements (C3D4) were employed to mesh the vertebral body, while hexahedral elements (C3D8H) were used for meshing the intervertebral disc. Shell elements were employed for meshing the cortical bone, cartilage of the facet joint, and endplate, with respective thicknesses of 1 mm, 0.6 mm, and 1 mm. The remaining structures were also meshed using shell elements. The intervertebral disc was divided into the nucleus pulposus and annulus fibrosus, with the nucleus pulposus accounting for 40% of the intervertebral volume. The annulus fibrosus comprised the annulus fibrosus matrix and fibres, with the fibres inclined at angles ranging from − 30° to + 30° relative to the horizontal plane. The model included seven ligaments, namely the ALL, PLL, LF, ISL, SSL, TL, and CL, which were modelled as tension-only beam elements (T3D2). Frictionless sliding was assumed for the facet joint contact. The material properties used in the finite element model were obtained from relevant literature. Detailed material properties for each element are presented in Table 1.

**Table 1:** Material properties used by finite element mode

Component	Element type	Young’s modulus (MPa)	Poisson ratio	Crosssectional area (mm^2^)
*Vertebra*				
Cortical bone	C3D4	12,000	0.3	
Cancellous bone	C3D4	100	0.2	
Posterior element	C3D4	3500	0.25	
Sacrum	C3D4	5000	0.2	
Facet	C3D4	11	0.2	
Disc	–	–	–	
Endplate	C3D8R	24	0.4	
Nucleus pulpous	C3D8RH	1	0.49	
Annulus ground substance	C3D8RH	2	0.45	
Annulus fbre	T3D2	360–550		0.15
*Ligaments*				
ALL	T3D2	7.8		63.7
PLL	T3D2	10		20
LF	T3D2	15		40
CL	T3D2	7.5		30
ISL	T3D2	10		40
SSL	T3D2	8		30
ITL	T3D2	10		1.8
Implants	C3D4	110,000	0.3	

*ALL*—Anterior longitudinal ligament; *PLL*—Posterior longitudinal ligament; *LF*—Ligamentum favum; *CL*—Capsular ligament; *ISL*—Interspinous ligament; *SSL*—Supraspinal ligament; *ITL*—Intertransverse ligament

### Construction of the finite element model

**Fig. 1:**
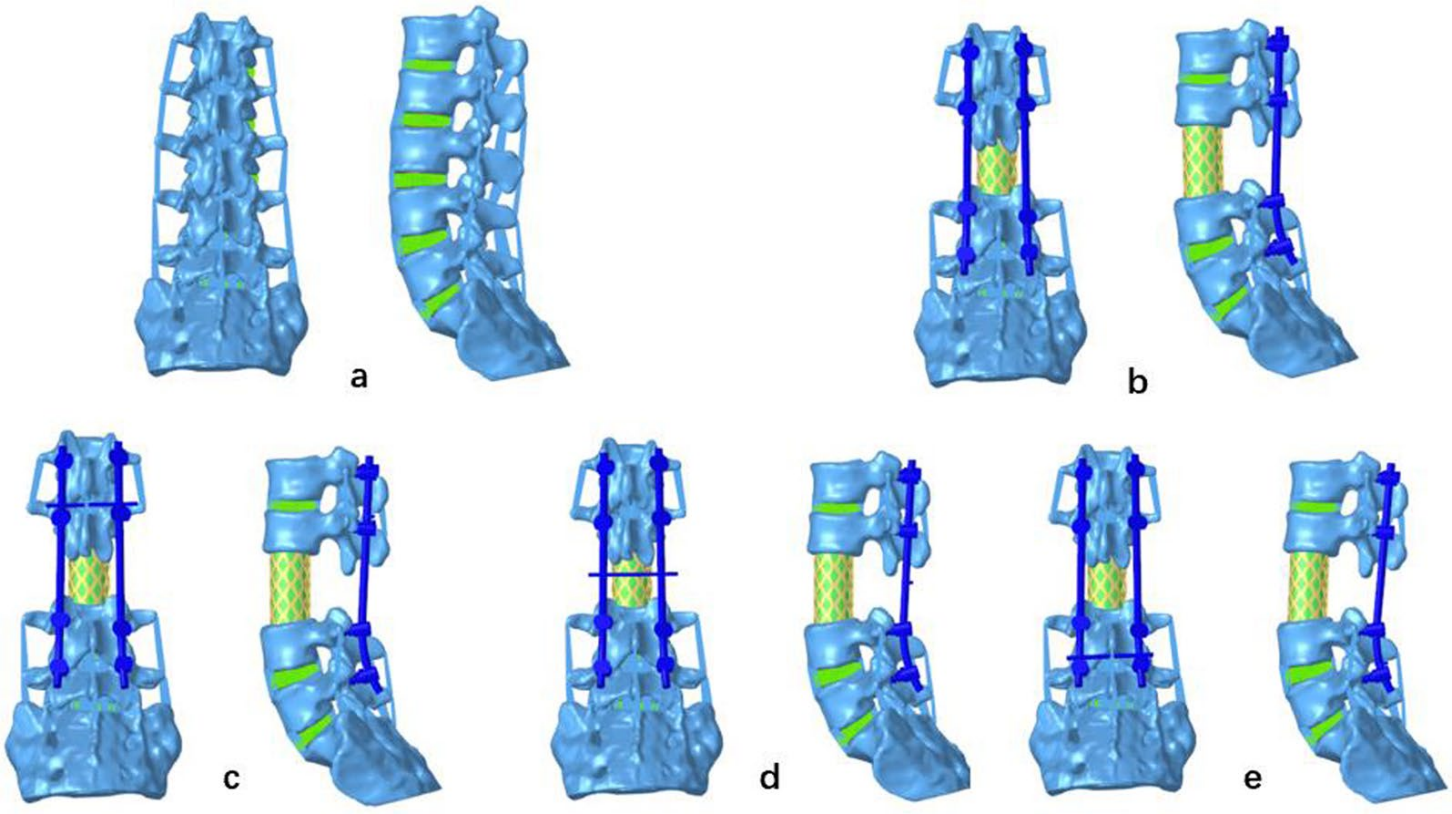
Anterior–posterior and lateral views of the five finite element models. Model **a**: complete model (lumbar [L] 1-sacrum); model **b**: total en bloc spondylectomy (TES) model without a transverse connector (TC); model **c**: TES model with a TC at L1–2; model **d**: TES model with a TC at L2–4; model **e**: TES model with a TC at L4–5

The L3 vertebral body, including the entire vertebral body, superior and inferior endplates, annulus fibrosus and nucleus pulposus, and ALL, was completely removed to simulate the TES surgical procedure. A titanium cage, with a length equivalent to that of the original vertebral body and a diameter of 25 mm, was used to replace the excised vertebral body. Fixation of the vertebral pedicle screws was performed at L1–2 and L4–5, with a diameter of 6.0 mm and a length of 45 mm. Five models were constructed, namely the complete model (L1-S), the TES model without a TC, the TES model with a TC at L1–2, the TES model with a TC at L2–4, and the TES model with a TC at L4–5 (Fig. 1).

### Boundary and loading conditions

The S1 vertebral body was immobilised in all six degrees of freedom. An 8 N/m torque was applied at the central point on the centreline connecting all the vertebral bodies to simulate anterior flexion. A 6-N/m torque was applied to simulate posterior extension, a 6-N/m torque was applied to simulate lateral bending, and a 4-N/m torque was applied to simulate axial rotation [12].

## Results

### Validation of model effectiveness

**Fig. 2:**
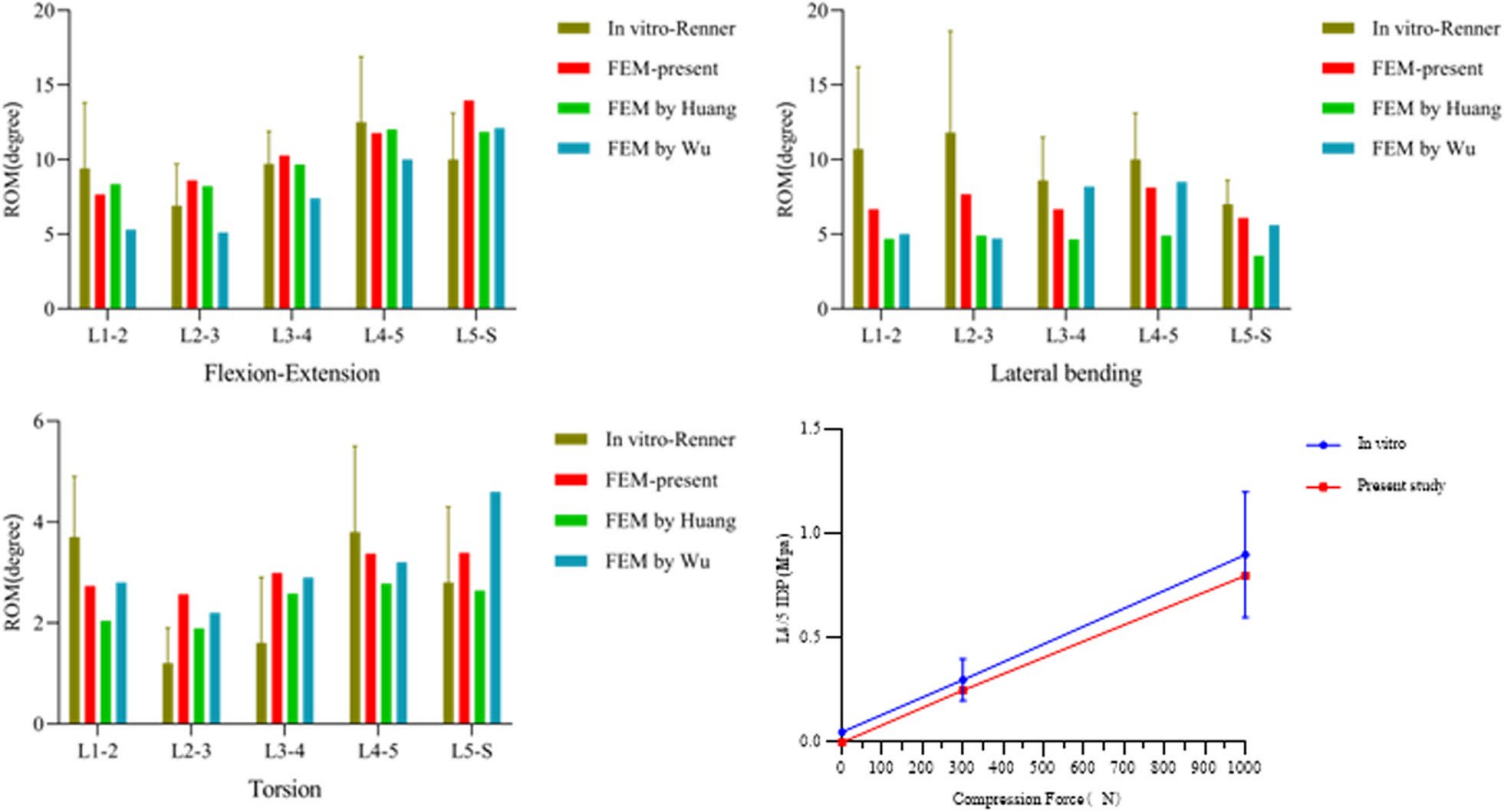
Comparison of the range of motion (ROM) and lumbar 4/5 intervertebral disc pressure between the present study, previous in vitro studies, and finite element models

The complete L spine model was compared with previous finite element models and in vitro experiments to measure the ROM in flexion, extension, lateral bending, and axial rotation. By applying identical loads and boundary conditions, the overall and segmental ROM of the L spine, as well as the intervertebral disc pressure at L4–5, were quantified. The obtained results demonstrate a favourable agreement between the finite element model and previous in vitro experiments and FEAs in terms of L spine ROM. Furthermore, a consistent trend of increased intervertebral disc pressure at L4–5 was observed [12–15]. Therefore, the validity of the finite element model for the L spine was confirmed. Detailed results are presented in Fig. 2.

### Comparison of the overall ROM between the different models

**Fig. 3:**
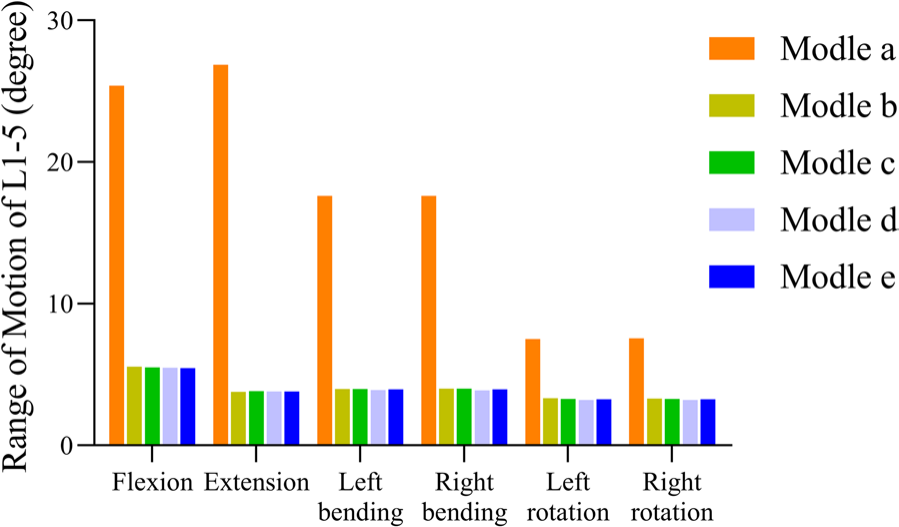
Changes in the range of motion at lumbar 1–5 for different models

Upon fixation of all segments, the overall ROM of the fixed segments (L1–5) significantly decreased compared with the complete model, indicating an effective stabilisation effect of the internal fixation device. Notably, the internal fixation device exhibited the highest degree of constraint in terms of overextension (95–96%), followed by flexion (92%) and left/right lateral bending (85–86%), while exhibiting relatively less restriction on left/right rotation (54–56%). Interestingly, the overall ROM showed no significant differences between models with or without TCs and across different TC positions, with minimal variations of < 0.11° and a reduction of 3%. The relevant results are presented in Fig. 3.

### Stress of the internal fixation system in different models

**Fig. 4:**
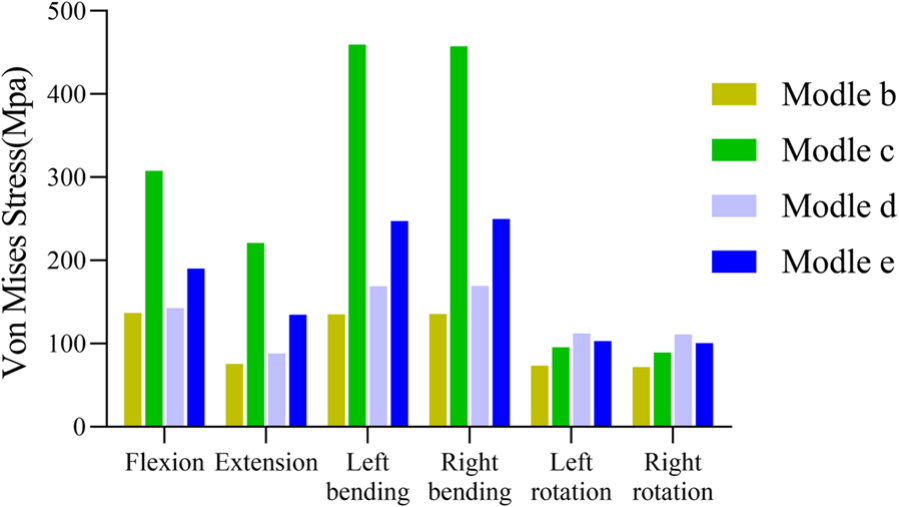
Maximum von Mises stress (MPa) for pedicle screw-rod systems in different models

**Fig. 5:**
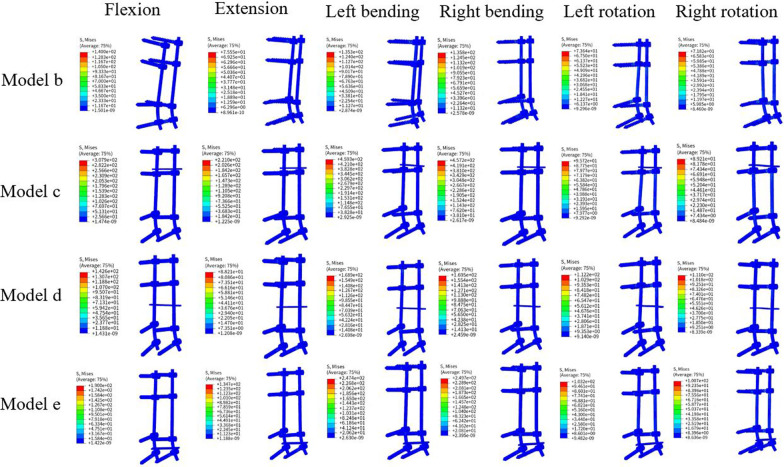
The von Mises stress distribution of the pedicle screw-rod systems in different models

In the model without a TC, the pedicle screw-rod system exhibited the highest maximum von Mises stress (VMS) during anterior flexion, reaching 136.9 MPa, while the lowest stress was observed during right rotation, reaching 71.8 MPa. However, upon the addition of a TC, the maximum VMS of the pedicle screw-rod system increased to varying degrees. In the model with a TC placed at L1–2, the maximum VMS was 459.3 MPa, representing a 336% increase in stress. Similarly, the model with a TC placed at L2–4 exhibited a maximum VMS of 169.5 MPa, corresponding to a stress increase of 124% increase in stress. In the model with a TC placed at L4–5, the maximum VMS reached 249.7 MPa, resulting in a stress increase of 182%. The stress variations in the pedicle screw-rod systems are presented in Figs. 4 and 5.

### Stress distribution of the titanium cage in different models

**Fig. 6:**
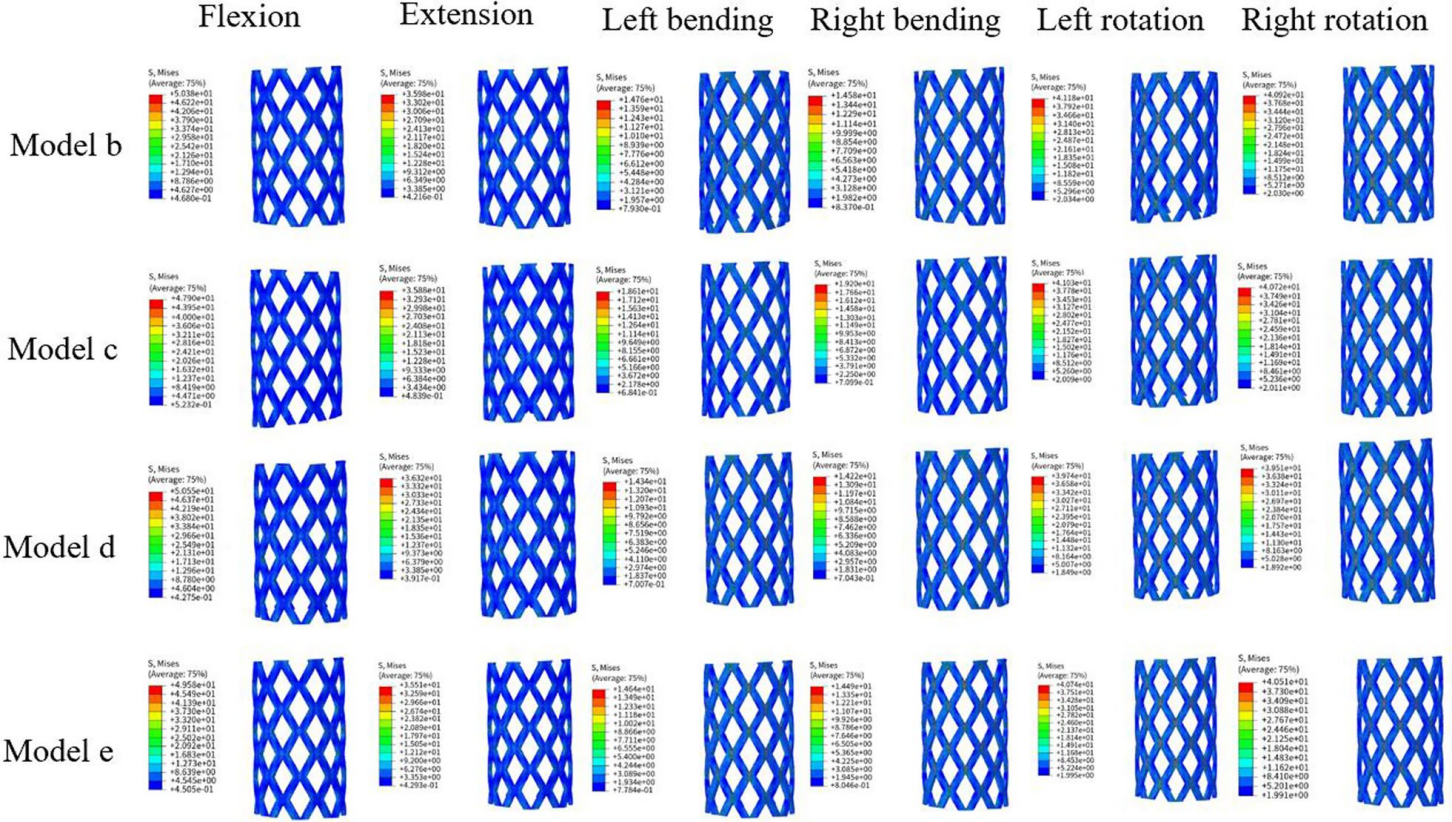
The von Mises stress distribution of titanium cages in different models

The stress variation of the titanium cage was not significant, regardless of the presence of TCs. Among all the models, the highest stress was observed during spinal flexion, particularly in the model with a centrally placed TC, reaching 50.55 MPa. The lowest stress was observed during lateral bending in model 3, which had a superiorly placed TC, with a stress level of 14.2 MPa. The stress distribution remained relatively unchanged with the application of TCs during flexion, extension, lateral bending, or rotation. The most notable change was observed during flexion, with a 5% reduction in stress within the titanium cage when TCs were used. The smallest change was observed during extension, with a 1% reduction in stress within the titanium cage when TCs were used. Details regarding the specific stress distribution are presented in Fig. 6.

### VMS adjacent to the endplate

**Fig. 7:**
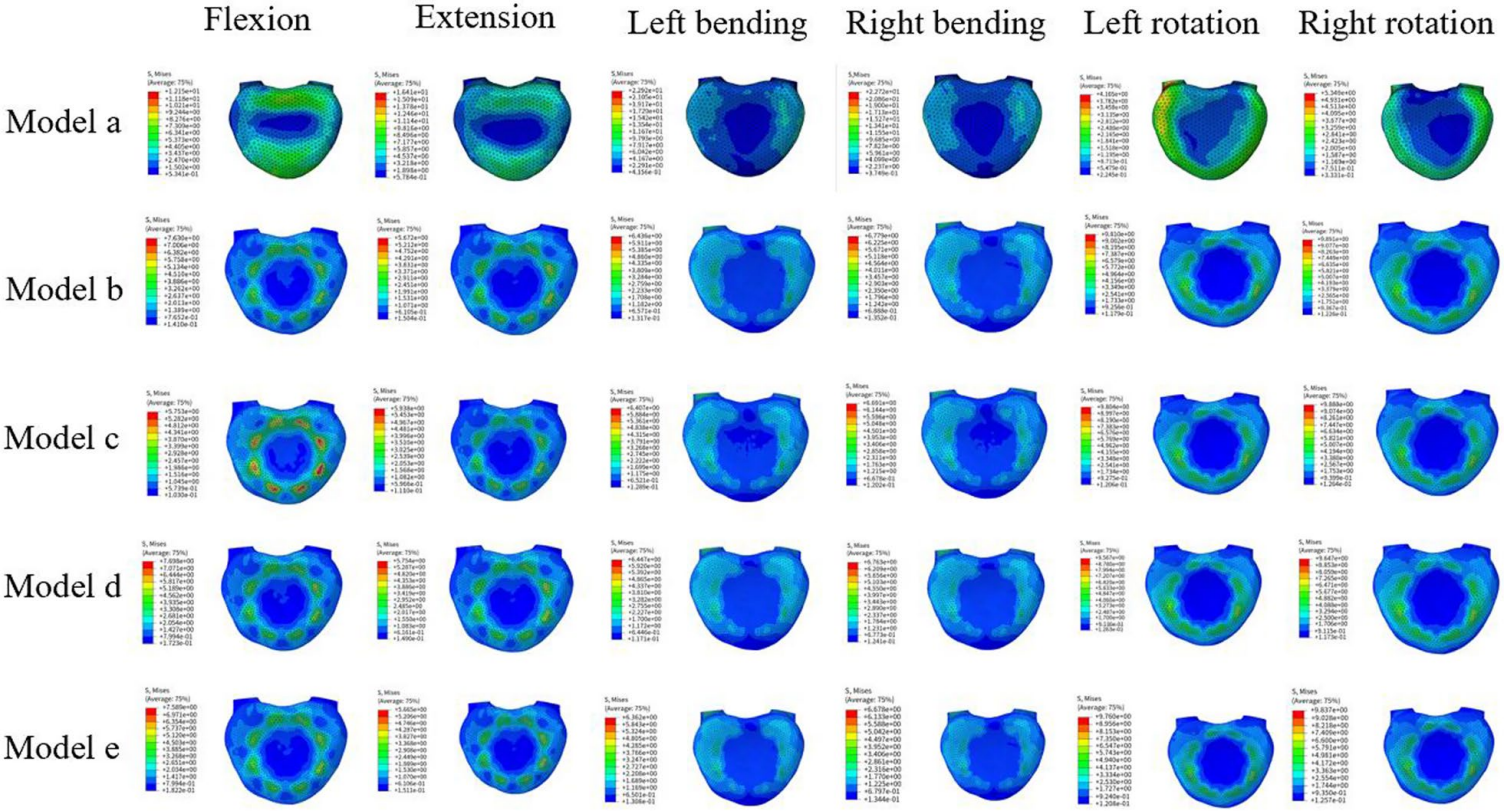
The von Mises stress distribution of the inferior endplate at lumbar 2

The stress levels on the inferior endplate at L2 were the highest during rotation in the absence of TCs, with a maximum stress occurring during rotation at 9.85 MPa, while the lowest stress was observed during extension at 5.67 MPa. Upon introducing TCs, a reduction of 25% in stress during flexion was observed in the model with a TC placed at L1–2. However, minimal changes were observed during extension, lateral bending, and rotation. In models with TCs placed at other positions, there were no significant changes in stress levels on the inferior endplate of L2. A graphical representation of these stress changes is presented in Fig. 7.

**Fig. 8:**
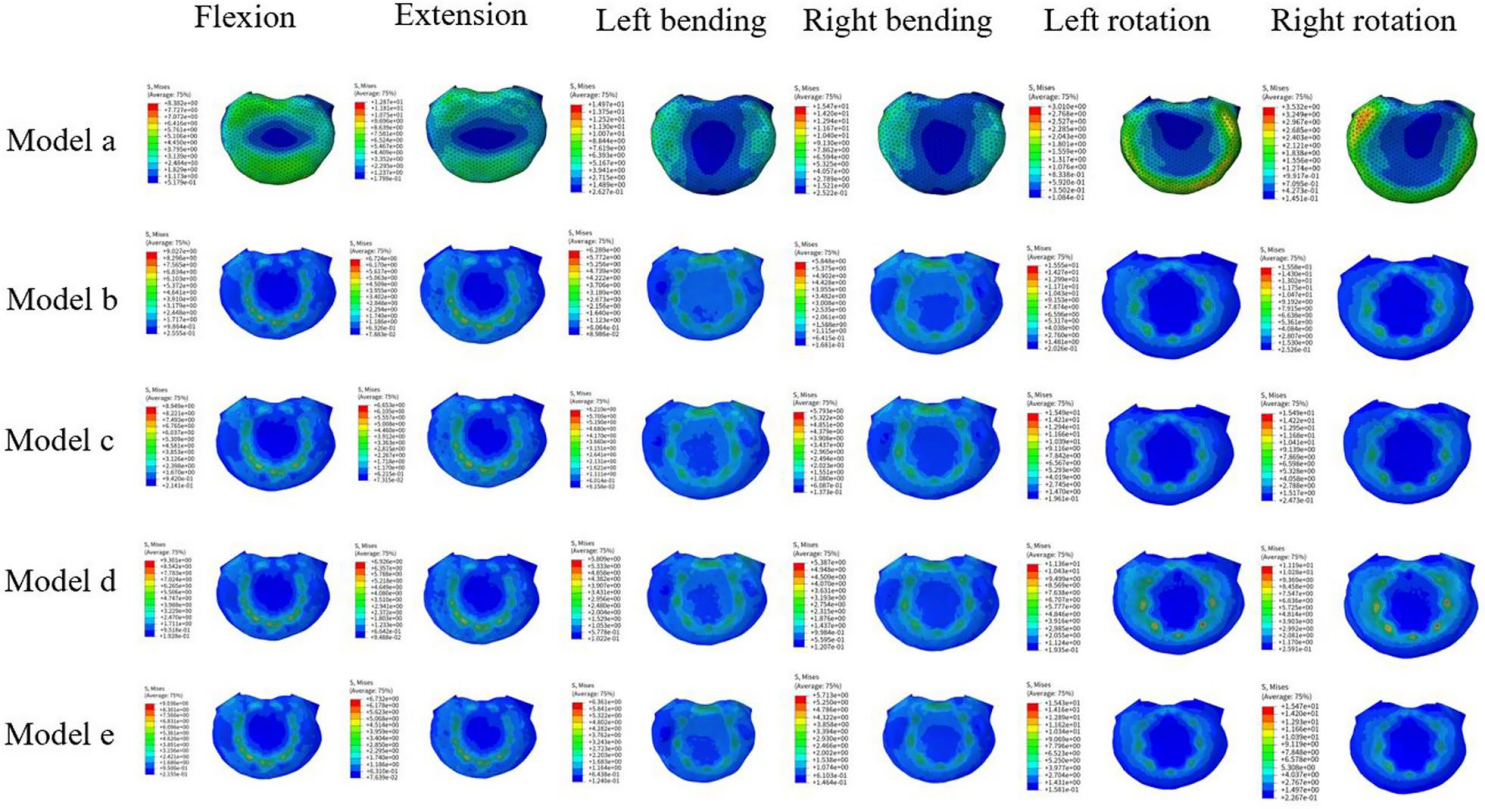
The von Mises stress distribution of the superior endplate at lumbar 4

In the absence of TCs, the stress on the superior endplate at L4 was the highest during rotation, with a maximum stress occurring during rotation at 15.57 MPa, while the lowest stress occurred during lateral bending at 6.07 MPa. Upon introducing TCs, the change was observed in the model with a centrally placed TC, with a reduction of 28% in stress during rotation. However, minimal changes were observed during flexion, extension, and lateral bending. Models with TCs placed at other positions did not exhibit major changes in stress levels on the superior endplate at L4. The specific stress changes are presented in Fig. 8.

## Discussion

The TES technique is widely used for the complete resection of primary or secondary spinal tumours, with studies demonstrating excellent clinical outcomes [16–19]. However, due to the complete removal of the anterior vertebral body and posterior vertebral arch, the stability of the spine is significantly compromised. Consequently, achieving spinal stability poses a substantial challenge in TES surgery, and internal fixation failure emerges as the most common complication [20]. The reported incidence of screw and rod fractures following TES is 26.3–40%, and implant subsidence also presents a considerable concern. For instance, Matsumoto et al. reported internal fixation failure in six out of 15 TES cases, with an average occurrence time of 28.3 months [4]. In a follow-up study by Kwon et al. [21] mechanical failure was observed in three out of 19 patients with primary tumours (33%) and two patients with metastatic tumours (20%). In Liu et al. [16] study, internal fixation breakage was observed in six out of 18 patients with giant cell tumours. Internal fixation failure could result in symptoms such as local pain and neurological dysfunction, significantly affecting patient well-being. Exploring the biomechanical changes of spinal internal fixation following TES could provide surgeons with a better theoretical basis, leading to a reduction in postoperative complications and the need for revision surgery. Internal fixation failure occurs when stress concentrations exceed the load-bearing capacity of the fixation device. Therefore, it is crucial to develop a reliable stabilisation method that offers sufficient stability while minimising stress concentration. TCs have been recognised for improving vertebral stability by connecting the pedicle screw-rod systems on both sides. Although some reports suggest that TCs might lead to stress concentration and increase the likelihood of screw and rod fractures, limited research exists on the biomechanical effects of TCs in TES. Our study aimed to investigate the application of TCs in TES through a series of relevant experiments to bridge this gap and to provide guidance for surgical planning. Five models were designed for the trial, namely the complete model, the L3 vertebral TES model (without a TC), the L3 vertebral TES model with L1/2 TC placement, the L3 vertebral TES model with L2/4 TC placement, and the L3 vertebral TES model with L4/5 TC placement. This study evaluated the biomechanical characteristics of different models.

It is important to acknowledge that biomechanical studies cannot simulate successful intervertebral fusion between vertebrae. According to the definition by the Food and Drug Administration, intervertebral fusion can be considered present when there are connected bone trabeculae between segments, allowing for translational mobilisation of < 3 mm and ROM of < 5° [4]. In our study, the ROM of all adjacent segments in the postoperative models was < 5°, indicating effective fixation and the potential for favourable intervertebral fusion. Previous studies have suggested that only an outcome difference > 20% could be considered significant [4, 22]. In our model, the maximum difference observed in the ROM of the fixed segments was 3.29%, which was significantly less than the 20% threshold. This observation holds regardless of whether TCs were used or their placement position. Consequently, it can be concluded that there is no significant difference in the ROM of the fixed segments between cases with and without TC placement. Previous studies have suggested that TCs can limit the rotational ROM. Peltier et al. [23] conducted experiments on thoracolumbar segment models using short-segment fixation and found that the application of two TCs increased torsion stiffness but did not increase stiffness in flexion, extension, and lateral bending. Similarly, Cornaz et al. [24] demonstrated that TCs had minimal impact on the ROM values of the fixed segments in single-segment cortical bone trajectory screw fixation. They mainly reduced stresses during partial angular rotation, suggesting that TCs might not be necessary in such cases. The findings of our study do not entirely align with these previous studies, which could be attributed to the differences in the fixed segments examined. TES surgery involves long-segment fixation, which provides significantly greater stability between two adjacent vertebrae compared with short-segment fixation between a single vertebra and its adjacent counterparts. Consequently, TCs might not offer additional segmental stability in long-segment fixation. Furthermore, it is worth noting that our study focused on L segments, while previous studies often investigated thoracic or thoracolumbar segments. This disparity in segment selection may also contribute to the discrepancies observed.

Fracture of the pedicle screw-rod system is a common complication after TES. For instance, in a study by Park et al. [6] out of 32 patients who underwent TES, 12 patients experienced screw and rod fractures. Similarly, Shimizu et al. [25] reported internal fixation failure in 44 (32.4%) of the 136 patients they followed up, with an average occurrence time of 31 months. Stress concentration within the internal fixation is recognised as one of the factors contributing to such fractures. Previous studies have indicated that the use of TCs might lead to stress concentration, thereby increasing the likelihood of pedicle screw-rod system fracture. In an FEA study, Hong et al. simulated the pedicle subtraction osteotomy (PSO) at L4 and observed that TC placement within two segments of the bone-cutting site resulted in stress concentrations. They found that the application of TCs increased the maximum VMS of the rod by 283.3% and 247.6% during spinal flexion and extension, respectively. Their study concluded that placing TCs farther away from the two segments had a less impact on internal fixation stresses [22]. Similarly, Park et al. [11] using FEA to simulate L4 vertebral PSO discovered that TCs increased stress on the rod, particularly during flexion and extension, potentially leading to internal fixation device fracture. The above-mentioned study primarily focused on measuring stresses on the screw and rod of the L spine during flexion and extension, neglecting the analysis of lateral bending and rotation. Our study also investigated this aspect and yielded similar findings. In cases where TCs were not applied and only pedicle screws were used for fixation, the maximum VMS of the pedicle screw and rod observed during anterior flexion, posterior extension, right and left lateral bending, and rotation were 136.9 MPa, 75.5 MPa, 135.8 MPa, and 73.6 MPa, respectively. However, when TCs were incorporated for fixation, a significant increase in the maximum VMS was observed. Specifically, during anterior flexion, posterior extension, left and right lateral bending, and rotation, the maximum VMS values were 307.9 MPa, 221 MPa, 459.3 MPa, and 112.2 MPa, respectively. These values represented substantial increases by 224.9%, 292.5%, 338.2%, and 152.4%, respectively. Notably, the greatest increase in VMS was observed during lateral bending, reaching 338.2%. These findings indicate that the implementation of TCs led to stress concentration, which could potentially result in screw and rod fractures and internal fixation failure.

Prosthesis subsidence is a commonly encountered complication following TES. Based on a previous report, the fracture strength of cortical bone ranges from 90 to 200 MPa [26]. Prosthesis subsidence might occur when the contact stress between the prosthesis and the bone exceeds 90 MPa. In cases where only pedicle screws were used for fixation after TES, the maximum VMS stress on the superior endplate at L2 was 9.89 MPa, observed during vertebral rotation. Upon incorporating TCs for fixation, the stress on the inferior endplate at L2 did not exhibit significant changes, with the maximum VMS being 9.89 MPa during rotation. Similarly, without TCs, the maximum stress on the superior endplate at L4 was 15.58 MPa during vertebral rotation. With the addition of TCs for fixation, the stresses on the superior endplate at L4 did not demonstrate significant alterations, with the maximum VMS still occurring during rotation at 15.49 MPa. These findings indicate that the application of TCs did not effectively reduce the stresses on the superior and inferior endplates. Importantly, in all models, the stresses observed in the superior and inferior endplates remained below 90 MPa, which is significantly lower than the strength required to cause cortical bone damage. Therefore, the fixation with pedicle screws and rods can effectively stabilise the vertebral body, rendering the use of TCs unnecessary.

Previous studies have also questioned the efficacy of TC application. Garg et al*.* analysed the use of the pedicle screw-rod systems in patients with adolescent idiopathic scoliosis and concluded that TCs did not yield additional clinical or imaging benefits. They suggested reducing the use of TCs to save medical costs without compromising prognosis [27]. Similarly, Dhawale et al. [28] arrived at a similar conclusion, observing no significant effects of TCs on the main Cobb angle and main correction percentage in their comparison study. It is crucial to consider the complications associated with TCs in clinical practice. Kim et al. [29] studied adult patients with scoliosis and found that 69% of them had TCs placed in the pseudojoint positions. Additionally, How et al. [30] identified TC application as a risk factor for pseudojoint formation in orthopaedic surgery for spinal deformities. Rahmathulla et al. [31] reported that TCs might contribute to spinal stenosis and delayed cerebrospinal fluid leakage. In our study, the use of TCs in TES did not significantly affect the ROM in the fixed segment or generate greater stress at the adjacent endplate. However, it did significantly increase the maximum stress experienced by the pedicle screws and rods, which might increase the risk screw and rod fracture and surgical failure. Therefore, routine TC placement in TES is not recommended.

This study has certain limitations. First, the finite element model data used in this study were obtained from a male volunteer, aged 21 years, which might limit the generalisability of the findings. Additionally, the absence of statistical analysis is a common limitation of FEA. Furthermore, the finite element model employed in this study was simplified, and the assumption of isotropic material properties for individual structures might not accurately reflect the biomechanical changes of the L spine. Lastly, the validation of our finite element model was based on comparisons with previous studies rather than on cadaveric models, potentially affecting the validity of the results. Future research should aim to address these limitations by conducting systematic and comprehensive biomechanical studies to further validate our conclusions.

## Conclusion

The application of TCs in TES does not yield substantial enhancements in fused segment stability or stress reduction on adjacent endplates. Conversely, it elevates the maximum VMS stress on internal implants, increasing the risk of screw and rod fracture and consequently resulting in surgical failure. Based on the findings of the present study, the routine use of TCs in TES is not recommended.

## Data Availability

The datasets used and/or analysed during the current study available from the corresponding author on reasonable request.
